# Hallucinations in the months after a trauma: An investigation of the role of cognitive processing of a physical assault in the occurrence of hallucinatory experiences

**DOI:** 10.1016/j.psychres.2016.10.081

**Published:** 2016-12-30

**Authors:** Georgina Geddes, Anke Ehlers, Daniel Freeman

**Affiliations:** a Department of Psychiatry, University of Oxford, Warneford Hospital, Oxford OX3 7JX, UK; b Department of Experimental Psychology, Tinbergen Building, 9 South Parks Rd, Oxford OX1 3UD, UK

**Keywords:** Post-traumatic stress disorder, PTSD, Re-experiencing, Peri-traumatic cognitions, Intrusive memories, Post trauma cognitions

## Abstract

The role that cognitive processing of a recent trauma has in the occurrence of hallucinations has not been examined longitudinally. This study investigated trauma-related cognitive predictors of hallucinations in the months following an interpersonal assault. Four weeks after treatment at an emergency department for interpersonal assault injuries, 106 participants were assessed for peri-traumatic cognitive processing, cognitive responses to trauma memories, negative beliefs about the self, Posttraumatic-stress disorder (PTSD), and hallucinatory experiences. Hallucinatory experiences were reassessed six months later. Cognitive processing during trauma (lack of self-referential processing, and dissociation), beliefs about permanent negative change, self-vulnerability, and self-blame and cognitive response styles (thought suppression, rumination, and numbing) were significant predictors of later hallucinations. The way in which trauma is processed may partly determine the occurrence of hallucinations.

## Highlights

• The first longitudinal investigation of trauma-related cognitive predictors of hallucinations.
• A range of peri- and post traumatic processing predicted later hallucinatory experience.
• The way in which trauma is processed may partly determine hallucination occurrence.
• The main weakness of the study was that it was a secondary analysis of an existing dataset.

## 1 Introduction

Many clinical researchers consider childhood trauma as a possible contributory factor in the occurrence of hallucinations, based upon substantial evidence of an association found in retrospective studies (e.g. Read et al., 2005). An alternative, though complementary, perspective suggests that exposure to more proximal ‘life events’, recent negative occurrences that bring about a substantial change in personal circumstances for the worse, increases the risk in the subsequent weeks of psychotic experiences such as hallucinations (for review see Beards et al., 2013). The implication of trauma in the occurrence of hallucinations has led to cross-sectional studies of trauma-related processes and hallucinations (e.g. Gracie et al., 2007; Morrison and Petersen, 2003). Cognitive processing related to recent trauma in the prediction of subsequent hallucinations has not yet been tested. This paper reports such a test: examining cognitive processing in the aftermath of a recent physical assault in relation to the occurrence of hallucinations.

### 1.1 Cognitive processing during and in the aftermath of trauma

Cognitive processes during and after trauma have been extensively examined as factors in the development and persistence of post-traumatic stress disorder (PTSD) (e.g. Dunmore et al., 1999; Ehring et al., 2008; Kleim et al., 2007). At the centre of Ehlers and Clark's (2000) model is the idea that PTSD occurs when individuals process the trauma in a way that leads to a sense of current threat. This is thought to result from a combination of problematic information processing during trauma and negative appraisals of the trauma and its consequences. Problematic peri-traumatic cognitive processing, such as predominance of data-driven processing (focussing on sensory impressions), lack of self-referential processing (i.e. insufficient linking of the event to other autobiographical knowledge) and peri-traumatic dissociation results in a poorly elaborated and poorly contextualised memory of the event. As such, these memories are easily and involuntarily triggered into consciousness and have a strong sense of occurring in the present (‘nowness’, Michael et al., 2005). PTSD is thought to persist because negative appraisals of the trauma and its effects motivate a series of maintaining cognitive and behavioural strategies; these include cognitive responses to the intrusive memories such as rumination and thought suppression (Ehlers and Clark, 2000), persistent dissociation (Murray et al., 2002) and excessive precautions (safety behaviours).

### 1.2 Understanding trauma and hallucinations

A number of ways that trauma may lead to hallucinations have been proposed, five of which are highlighted here. First, the phenomenological overlap between hallucinations and the intrusive images and ‘flashback’ experiences often considered the hallmark symptoms of PTSD (Ehlers and Steil, 1995) has been noted, leading to suggestions that a minority hallucinations may be simply better understood as re-experiencing phenomena (e.g. McCarthy-Jones and Longden, 2015). Second, a ‘weaker’ version of the first account is that trauma related intrusive cognitions, including poorly contextualised trauma memories, are the type of low cognitive effort mental events that are most prone to source monitoring errors (e.g.Larøi et al., 2004) and hence lead to an increase in the occurrence of hallucinatory experience. Third, having been found to positively mediate the effect of childhood trauma on hallucination proneness (Varese et al., 2012), dissociation has been considered one explanatory factor. Dissociation may be particularly relevant to hallucinations as it is characterised by a sense of disconnect from reality. However the mechanism through which dissociation might promote hallucinations remains to be clarified. Fourth, exposure to trauma can cause negative changes in thoughts about the self (e.g. Foa et al., 1999), such as the sense of being permanently changed for the worse (e.g.Dunmore et al., 2001), resulting in previous conscious content being experienced as alien (e.g. Morrison, 2001). Finally, maladaptive cognitive responses to intrusive memories and thoughts (e.g. rumination and thought suppression), may amplify the chances of hallucinatory experiences as they not only increase the occurrence of intrusive cognitive events but also increase their intensity (Guastella and Moulds, 2007, Yoshizumi and Murase, 2007).

### 1.3 Current study

In this study, hallucinations are examined in relation to the processing of a recent interpersonal assault. This secondary analysis of a study focussing primarily on paranoia (Freeman et al., 2013) aimed to investigate associations between trauma-related processing and cognitions, derived from Ehlers and Clark's (2000) model of PTSD, and hallucinations. It was hypothesised that problematic peri-traumatic processing (data-driven processing, lack of self-referential processing and dissociation), negative appraisals of the consequences of trauma (permanent change, vulnerable self, self-blame) and maladaptive cognitive control strategies (thought suppression, rumination and numbing) would all predict the presence and maintenance of hallucinations.

## 2 Method

For a full description of methods see Freeman et al. (2013). The method used paralleled that of prospective studies of PTSD after assault previously conducted (e.g. Dunmore et al., 2001; Kleim et al., 2007). All individuals attending A&E Department for injuries relating to an interpersonal assault were written to and invited to take part in the study. Participants had an initial assessment between 4 and 6 weeks after the assault and were followed up over the following six months.

### 2.1 Participants

106 individuals were recruited. In order to meet the inclusion criteria individuals must have experienced a distressing assault within the previous month, attended the Accident and Emergency (A&E) Department at King's College Hospital, London for related injuries, been aged 18–65 years, and been able to attend a baseline assessment between 4 and 6 weeks after the assault. The main exclusion criteria were that the assault was part of on-going abuse, that there was a history of diagnosed severe mental illness, that they had a diagnosed alcohol or drug dependence, or that they had insufficient command of English so that the assessments could not be completed.

Broadly the types of assault experienced were confrontations (n=33), random attacks (n=24), muggings (n=22), one-off attacks from family member or friends (n=19) and attacks in the context of work (n=8). All participants reported sustaining injuries during the assault.

### 2.2 Measures

The subset of study variables used in this analysis are summarized below.

Hallucinatory experience at baseline was assessed with the self-report Cardiff Anomalous Perceptions Scale (CAPS) (Bell et al., 2006) and the interviewer-rated hallucinatory behaviour item of the Positive and Negative Symptoms Scale (PANSS) (Kay et al., 1987). At the 6 month follow up only the hallucinatory behaviour item of the PANSS was used. The interrater reliability of the two assessors (postgraduate psychologists) for the PANSS positive items was assessed using 12 audiotapes of the assessments. The intra-class correlation coefficient (0.92) indicated very high levels of reliability.

PTSD severity at baseline and at 6 months was measured with self-report Posttraumatic Diagnostic Scale (PDS) (Foa et al., 1999) and the interviewer versions of the PTSD symptom scale (PSSI) (Foa et al., 1993), combined with the PTSD section of the structured Clinical Interview for DSM-IV (SCID; First et al., 1996). Re-experiencing was assessed with the first 5 items of the PDS.

For assessment of peri-traumatic cognitive processing the Thoughts and Feelings During the Assault scale (Halligan et al., 2003, Halligan et al., 2002); Mental Defeat Scale (Dunmore et al., 1999) and Cognitive Processing Questionnaire (Halligan et al., 2003, Halligan et al., 2002) were used. Trauma appraisals were assessed with an updated version of the Posttraumatic Cognitions Inventory (Foa et al., 1999) and cognitive responses to trauma memories were assessed with the Response to Intrusions Questionnaire (Clohessy and Ehlers, 1999, Murray et al., 2002). Affective symptoms were assessed using the Depression Anxiety Stress Scales (Lovibond, 1995).

### 2.3 Statistical analysis

All analyses were carried out using SPSS version 13. A similar analytic strategy to Freeman et al. (2013) was used for comparability. The first stage was to provide a description of levels of hallucinatory experience within the participant group in the months following an assault. The patterns of correlation between PTSD re-experiencing and hallucinatory experience were then examined. The third stage used a series of simple univariate linear regressions to examine the prediction of hallucinatory experience at baseline and at 6 months. Finally predictor variables were assessed again after controlling for the baseline score of the dependent variable.

## 3 Results

### 3.1 Demographic details

There were more male than female participants (79 men and 27 women); the mean age was 34.4 years (S.D.=11.6 years).

### 3.2 Presence of hallucinations/anomalous experience

Mean scores of the hallucination and re-experiencing measures are displayed in Table 1.

There was a high correlation between the total scores for the self-report (CAPS) and the interviewer-rated (PANSS) hallucination assessments at baseline, *r=*0.54, *p<*0.001. Seven people (6.6%) were rated as having mild to moderate hallucinatory behaviour as measured by the PANSS. By the six month follow up there was a statistically significant reduction in hallucinatory behaviour as assessed by the PANSS hallucinatory behaviour item, t (92)=3.01, p=0.003. Of the 20 participants rated as having at least minimal hallucinatory behaviour at baseline, 16 had follow up data. Of these, 11 showed a decrease in their hallucinatory behaviour and 5 showed no change.

### 3.3 Hallucinatory behaviour and PTSD re-experiencing

Measures of re-experiencing at baseline positively correlated with baseline hallucinatory measures, CAPS: *r=*0.308, p=0.001; PANSS-hallucinations *r=*0.202, *p=*0.038. At six months there was a significant correlation between re-experiencing as assessed by items in the Posttraumatic Diagnostic Scale and hallucinatory behaviour as assessed by PANSS item, *r=*0.313, *p=*0.002. There was a statistically significant reduction in re-experiencing over the six months, t(93)=2.79, p=0.006.

### 3.4 Factors associated with the presence and persistence of hallucinatory behaviour

Table 2 provides the tests of the baseline variables predicting baseline hallucination scores. The majority of variables was associated with presence of hallucinatory behaviour as assessed by the PANSS item at baseline, and all correlated with self-reports of baseline hallucinatory behaviour (CAPS). Higher scores at baseline increased the likelihood of baseline hallucinatory behaviour.

Table 3 shows the ability of the individual baseline variables to predict hallucinatory experience as assessed by PANSS hallucination item at 6 months. Of the 12 trauma related baseline variables, 10 predicted the likelihood of hallucinatory experience at follow up. The individual predictor variables were assessed again after controlling for the baseline score of the dependent variable (i.e. to assess variable prediction above initial symptoms score). Even after controlling for baseline score of hallucinatory behaviour, 8 out of the 12 trauma related baseline variables significantly predicted hallucinations at 6 months.

## 4 Discussion

The study tested longitudinally the predictive relationship of cognitive processing of recent trauma to hallucinations. Whilst PTSD and, more recently, paranoia (Freeman et al., 2013), have been the focus of previous research, hallucinations in the months after an assault have not. The results of the exploratory study supported the hypothesis that particular styles of processing trauma (e.g. lack of self-referential processing, and peri-traumatic dissociation), negative appraisals of the consequences of trauma (e.g. permanent change, vulnerability, and self-blame), and maladaptive cognitive control strategies (e.g. thought suppression, rumination, and numbing) would all predict the presence and maintenance of hallucinations. How a trauma is processed may well impact on the likelihood of subsequent hallucination occurrence. This is, however, in the context that clear hallucinatory experience, such as hearing a voice, occurred infrequently in this non-clinical population and that the study design cannot rule out the influence of unmeasured confounders.

A new finding was the rates of hallucinatory experience at one month after assault and the reductions over time. Using the PANSS, 18.9% of participants were rated as having at least minimal hallucinatory experiences and 6.6% of participants having hallucinatory experiences rated as mild or moderate. There was a statistically significant reduction in hallucinatory behaviour between the baseline assessment and the six month follow up. One interpretation of these findings is that in the direct aftermath of trauma there is a raised likelihood of experiencing hallucinations. This increase in hallucinatory behaviour is, for the majority of participants, only temporary, and reduces over the following six months. This pattern also indicates that the higher rates of hallucinatory experience at one month were probably not simply a reflection of associations between high levels of hallucinatory behaviour and the likelihood of being a victim of assault.

The presence of hallucinatory experience allowed for the examination of the predictive ability of trauma-related processes derived from a cognitive model of PTSD (Ehlers and Clark, 2000). Findings suggest that trauma-related processing predicted the occurrence and maintenance of hallucinations. Re-experiencing, as measured by items 1–5 of the PDS, was found to correlate with all measures of hallucinatory experience, however, the correlation was only moderate, indicating that the hallucinatory experience scales were most likely not simply a measure of severe re-experiencing. This suggests that hallucinatory experience is unlikely to be just re-experiencing phenomena.

Certain peri-traumatic cognitive processing styles are linked with an increase in intrusive cognitions, individuals experiencing intrusive thoughts are at increased risk of developing hallucinations (Jones and Fernyhough, 2009). Both peri-traumatic dissociation and lack of self-referent processing were predictive of the occurrence and persistence of hallucinations. Whilst data-driven processing was a significant predictor of anomalous experiences as assessed by CAPS it did not significantly predict the presence or persistence of hallucinatory behaviour as assessed by the hallucinatory behaviour item on the PANSS. Whilst this is most likely due to methodological issues, it is interesting that it did not emerge as one of the stronger predictors as it has, like lack of self referent processing and peri-traumatic dissociation, been consistently linked with intrusive memories after trauma (Ehring et al., 2008, Halligan et al., 2003).

Post-trauma cognitions about a vulnerable self, permanent change and self-blame, along with mental defeat, predicted the presence of hallucinatory experience even after controlling for baseline hallucination symptom scores. These results highlight a potential role for negative appraisals of trauma and a sense of changed or defeated self in determining the occurrence of hallucinations. Mental defeat has previously also been shown to be one of the strongest predictors of PTSD after interpersonal trauma (Kleim et al., 2007). All three cognitive responses to trauma-related intrusions (rumination, thought suppression and numbing) included in this study also significantly predicted the persistence of hallucinatory experience. Thought suppression and rumination predicted later hallucinations, even after controlling for baseline hallucination score. It is possible that this is due to their impact on the frequency and intensity of intrusive cognitive events.

The main strength of this study was its longitudinal design. The prevalence of hallucinations provided the opportunity to examine the predictive ability of certain cognitive variables linked to trauma. However as this study is a secondary analysis of a study focussed upon paranoia, several limitations are apparent. It cannot be determined that the assault led to an increase in hallucinatory behaviour, it is likely that this could only be researched in a natural experiment. It may have been useful to have a measure of symptom severity at a time closer to the assault as it is possible that levels of hallucinatory behaviour may have already been reducing by 1 month. In addition a retrospective evaluation of pre-trauma hallucinatory behaviour might help support the supposition that the hallucinatory behaviour is a result of the assault. It is also worth noting that this study did not include sexual trauma, a type of trauma that has been specifically related to hallucinations (Read et al., 2003, Hardy et al., 2005).

The primary outcome measure, the PANSS item, is a very brief clinical tool for use in psychiatric settings and, as a result, it is insensitive to hallucinatory behaviour at the less severe end of the spectrum. Individual psychotic symptoms show quantitative variation, existing on a continuum with normal experiences. By extending the search for psychological mechanisms predictive of specific psychotic symptoms to samples at lower levels of the continuum (Garety and Freeman, 1999) our understanding of psychosis could potentially be enhanced (Johns and van Os, 2001). It would have been beneficial to include the CAPS in the 6 month assessment in order to give a more detailed account of hallucinatory behaviour at the lower levels of the continuum. It is important, when investigating how trauma reactions can include hallucinations, to consider both a) psychological processes which are common to PTSD and hallucinations and b) distinct psychological processes which are specifically associated with hallucinations. This may include source monitoring and reasoning biases. It would be useful to include measures of these in any future investigations of hallucinations in the direct aftermath of trauma.

## Figures and Tables

**Table 1:** Hallucination scores.

	**Baseline**		**6-month-follow-up**	
**Measure**	***n***	**Mean (S.D.)**	***N***	**Mean (S.D)**
PANSS (Hallucinatory behaviour)	106	1.26 (0.61)	93	1.11 (0.37)
CAPS	106	7.81 (6.56)		
PDS (*re*-experiencing items)	106	4.92 (3.81)	94	3.77 (3.9)

PANSS, Positive and Negative Symptom Scale; CAPS, Cardiff Anomalous Perceptions Scale; PDS, Posttraumatic Diagnostic Scale

**Table 2:** Individual baseline predictors of baseline hallucination scores.

	Baseline				
	**CAPS**			**PANSS**	
	Standard coefficient	*p*		Standard coefficient	*p*
PTSD					
PTSD severity	0.468	<0.001		0.277	0.004
PTSD- re-experiencing	0.308	0.001		0.202	0.038
Peri-traumatic cognitions					
Perceived threat to life	0.309	0.001		0.174	0.075
Negative emotions during trauma	0.212	0.029		0.168	0.084
Data-driven processing	0.191	0.050		0.071	0.473
Lack of self-referent processing	0.308	0.001		0.216	0.026
Dissociation	0.289	0.003		0.242	0.012
Mental Defeat	0.198	0.042		0.143	0.145
Cognitive responses to trauma memories					
Suppression	0.240	0.013		0.151	0.122
Rumination	0.256	0.008		0.186	0.056
Numbing	0.397	<0.001		0.256	0.008
Post trauma cognitions					
Vulnerable self	0.502	<0.001		0.391	<0.001
Permanent change	0.483	<0.001		0.375	<0.001
Self blame	0.349	<0.001		0.378	<0.001

PTSD, Post Traumatic Stress Disorder; CAPS, Cardiff Anomalous Scale; PANSS, Positive and Negative Symptom Scale

**Table 3:** Individual baseline predictors of hallucinatory behaviour (PANSS) at 6 month follow-up and adjusted for original PANSS hallucination symptom score.

	6 month			Controlling for baseline score of DV		
	Standard coefficient	*p*	r^2^	Standard coefficient	*p*	r^2^
Peri-traumatic cognitions						
Perceived threat to life	0.314	0.002	0.099	0.104	0.166	0.549
Negative emotions during trauma	0.168	0.107	0.028	0.021	0.772	0.539
Data-driven processing	0.160	0.125	0.026	0.072	0.318	0.544
Lack of self-referent processing	0.303	0.003	0.092	0.154	0.034	0.562
Dissociation	0.308	0.003	0.095	0.153	0.035	0.561
Mental Defeat	0.282	0.006	0.079	0.204	0.004	0.580
Cognitive responses to trauma memories						
Suppression	0.275	0.008	0.076	0.175	0.014	0.569
Rumination	0.298	0.004	0.089	0.181	0.011	0.571
Numbing	0.307	0.003	0.094	0.106	0.155	0.549
Post trauma cognitions						
Vulnerable self	0.464	<0.001	0.215	0.261	<0.001	0.592
Permanent change	0.398	<0.001	0.158	0.233	0.001	0.590
Self blame	0.344	0.001	0.118	0.177	0.015	0.569
